# Population genetics of cancer cell clones: possible implications of cancer stem cells

**DOI:** 10.1186/1742-4682-7-42

**Published:** 2010-11-09

**Authors:** Christopher T Naugler

**Affiliations:** 1 Department of Pathology and Laboratory Medicine, University of Calgary and Calgary Laboratory Services, C414, Diagnostic and Scientific Centre, 9, 3535 Research Road NW, Calgary AB, T2L 2K8 Canada

## Abstract

**Background:**

The population dynamics of the various clones of cancer cells existing within a tumour is complex and still poorly understood. Cancer cell clones can be conceptualized as sympatric asexual species, and as such, the application of theoretical population genetics as it pertains to asexual species may provide additional insights.

**Results:**

The number of generations of tumour cells within a cancer has been estimated at a minimum of 40, but high cancer cell mortality rates suggest that the number of cell generations may actually be in the hundreds. Such a large number of generations would easily allow natural selection to drive clonal evolution assuming that selective advantages of individual clones are within the range reported for free-living animal species. Tumour cell clonal evolution could also be driven by variation in the intrinsic rates of increase of different clones or by genetic drift. In every scenario examined, the presence of cancer stem cells would require lower selection pressure or less variation in intrinsic rates of increase.

**Conclusions:**

The presence of cancer stem cells may result in more rapid clonal evolution. Specific predictions from theoretical population genetics may lead to a greater understanding of this process.

## Background

Cancers have been referred to as microcosms of evolution [1,2]; different genetic clones within a given cancer mutate, compete and evolve resulting in the final outcome of a malignant neoplasm. A number of excellent recent reviews of this process have been published [1-4]. Because cancer cell clones can be thought of essentially as sympatric asexual species [3,5], theoretical population genetics as it applies to asexually reproducing organisms may provide additional valuable insights into the dynamics of cancer clonal evolution. With a few notable exceptions, however [5-9], largely missing from this discussion has been the inclusion of predictions from theoretical population genetics as they pertain to the population dynamics of asexually reproducing species. Such an approach is both timely and necessary as much remains to be elucidated about the population dynamics and evolutionary parameters of cancer cells [1].

The potential presence of cancer stem cells adds a further dimension to tumour clonal evolution. Cancer stem cells (a subpopulation of tumour cells with limitless replication ability and variable treatment resistance [10-17] have been described for a number of cancers, although the importance of cancer stem cells is still a matter of controversy. Cancer stem cells are often presented as an alternative model to tumour clonal evolution [12], but there is no reason that cancer stem cells may not themselves undergo clonal evolution [18].

In this paper I present several concepts from population genetics which have relevance to the population biology of tumour clones. I have not attempted to show the derivation of the equations presented here - interested readers should consult established reference sources if they require further information [19-22]. The specific issues I will to address are (1) how quickly can mutations favourable to the growth of a cancer accumulate in a given clone? (2) what are the mechanisms by which one cancer clone can replace another? and (3) how might these factors be affected by the presence of cancer stem cells?

## Results

### Accumulation of mutations favourable to the growth of a cancer

#### What mutations are "favourable" to a cancer cell?

Favourable mutations to a cancer cell could include mutations which enhance the growth potential of a cancer cell clone, aid in overcoming an anti-cancer defence mechanism, or aid in the competitive advantage of a given cell clone. Mutations favourable to the growth of a cancer cell may of course be detrimental to the ultimate survival of the host. As Merlo et al. [1] argued, "It might be that most genes in the human genome are devoted to building and maintaining a multicellular body, and are therefore irrelevant to a neoplastic cell under selection for increased survival and proliferation". In fact the shedding of "unnecessary" metabolic processes may even provide a further reproductive advantage for a cancer cell as it may reduce the diversion of energy away from cell division.

Although an individual cancer may have 11,000 different mutations [23], or perhaps orders of magnitude more [24], the number of significant genetic changes in carcinomas required to produce a malignant phenotype is estimated to be in the range of 3-7 [4,23,25,26]. So with respect to "favourable mutations", I am referring to this subset of genetically important mutations and not to the large number of background mutations.

#### Muller's ratchet

In sexually reproducing organisms like Humans, meiosis and natural selection work in tandem to remove deleterious mutations from the gene pool in the following way: meiosis randomly assorts the mutations among offspring and natural selection then removes the deleterious mutations through differential survival of those offspring. However as Muller pointed out nearly 80 years ago [27], asexually reproducing organisms lack a mechanism to remove deleterious mutations from the population. The corollary is that in asexual species there is also no mechanism to quickly combine favourable mutations in a single clone. For example, if three favourable mutations (*A*, *B *and *C*) all arose in a given sexually reproducing population, genetic recombination could assort these mutations into a single organism in a limited number of generations. However, in an asexually reproducing population, the only way for a single clonal line to possess all three mutations is if an individual already possessing one of the mutations (say, mutation *A*) then acquires mutation *B*, and finally a descendent of this clone then acquires mutation *C*. A general estimate of the number of generations necessary between the establishment of one favourable mutation and the establishment of a second favourable mutation in an sexual population is given by the equation ***G ***= ***log (N/s) ***[19], where ***G ***= the number of generations, ***N ***= population size, and ***s ***= the selective advantage of the new mutation. I will come back to selective advantage later. Of course, while it is true that mutations may be numerically more likely to arise in larger populations, the counterintuitive prediction is that such mutations may arise and become established more quickly in smaller populations. Table 1 shows that if we apply this equation to a series of hypothetical cancers of different sizes, we get the prediction that new favourable mutations will become established in a given cancer clone faster when the tumour is smaller. Likewise, for cancer stem cells, favourable mutations are predicted to become established at a faster rate in a stem cell model for any size of tumour but are predicted to be established most quickly in smaller cancers (Table 1)[28]. Several previous studies have reported an association between tumour heterogeneity and malignant potential [29,30]. The prediction of faster clonal evolution in smaller tumours could also mean that in the fragmented (i.e. smaller) subpopulations associated with genetic heterogeneity, new mutations may become established at a faster rate, thus adding to the malignant potential of genetically heterogeneic tumours.

**Table 1:** Number of generations for a favourable mutation to become established in a tumour cell clone [20].

Tumour Size (cm)	Number of cells	Estimated number of generations to establish a favourable mutation in a somatic cell clone	Estimated number of generations to establish a favourable mutation in a stem cell clone
0.1	4 189	6	1

0.5	523 599	8	3

1.0	4 188 790	9	4

2.0	33 510 322	10	5

3.0	11 3097 336	10	5

4.0	268 082 573	10	6

5.0	523 598 776	11	6

10.0	4 188 790 205	12	7

The results are rounded to the nearest whole number. The results were calculated by assuming a fairly weak selective advantage of 0.01 for the new clone. The number of cells as calculated by determining the number of cubes, 50 micrometers on a side, which would fill a sphere representing a tumour of a given size. The number of stem cells is calculated by assuming one stem cell per 57 000 non-stem cells [28]. A greater proportion of stem cells would result in a less dramatic difference.

Finally, if we consider a cancer stem cell model where clonal evolution is occurring only in a subset of the cancer cells (the stem cells) we can see that for any size of tumour, because of the much smaller number of stem cells, clonal evolution is predicted to be faster in stem cells compared to non-stem cells.

### What are the mechanisms by which one cancer cell clone can replace another?

The previous section dealt with the question of how quickly new favourable mutations may arise and become established. However, especially in large tumours, the time required for a new favourable mutation to occupy a large part of the tumour mass is also important [6]. So I will now turn to the question of how one clone can come to replace another within a tumour. Three basic mechanisms exist by which this may occur: genetic drift, variation in the growth rate (intrinsic rate of increase) of different clones and selection pressure favouring one clone over another.

#### Genetic drift

Recall that Muller's ratchet describes the situation where deleterious mutations may accumulate in an asexual population because of genetic drift as there is no mechanism to remove them. Conversely, favourable mutations could conceivably be aided in their increase by genetic drift but this effect may be difficult to disentangle from the other mechanisms outlined below. Accumulation of mutations by drift therefore is more relevant to mutations that are selectively neutral or deleterious to the cancer cell clone. For mutations that are deleterious, random drift will tend to fix a mutation if ***U/s > 1 ***[19] where ***U ***= the total number of mutations per genome and ***s ***= the selection pressure against individual mutations. Stoler et al. [23] estimated that there are at least 11,000 genomic alterations in the clone that generates a colon carcinoma. Other comparative work has suggested that approximately 25% of amino acid replacements destroy the resulting protein function [31], indicating that many of the mutations observed in cancer cells may be deleterious. The presence of such a large number of mutations (or even more by another estimate [32]) suggests that many mutations, even ones which are deleterious to the cancer clone, will accumulate and increase to fixation by random drift alone. Furthermore, as the number of mutations is likely much higher than the selection pressure against any given mutation, we would expect that even some mutations which are highly selected against will increase in the tumour. Finally as the number of mutations in a given cancer increase in the later stages of clonal evolution, we could also make the prediction that genetic drift may be more important in the later stages of a tumour, because of the increased number of mutations, despite the fact that the absolute number of cancer cells is also larger.

#### Variation in the intrinsic rate of increase

The rate at which a population increases is known as its intrinsic rate of increase and is described by the equation ***dx/dt = rx ***[20] where ***dx ***is the change in the number of individuals over time, ***dt ***is the time interval, ***r ***is the intrinsic rate of increase and ***x ***is the starting population size. Another way to think of ***r ***is as the 'compound interest' on the increase in population size. Consider the situation where two cancer clones exist within a tumour. They do not directly compete with each other (i.e. there is no selection) but they do differ in the speed with which they divide (their intrinsic rate of increase). In this situation, the clone with the higher intrinsic rate of increase will eventually overtake the slower growing clone even in the absence of any selection pressure. For two clones (***A ***and ***a***) with overlapping generations the rate of change (expressed as the change in proportion of ***A ***to ***a ***is given by the equation ***G = [ln (P***_***t***_***/(1-P***_***t***_***) - ln (P***_***0***_***/(1- P***_***0***_***)]/(r***_***A ***_***- r***_***a***_) [22] where ***G ***= the number of cell generations, ***P***_***0 ***_= the starting proportion of the two clones, ***P***_***t ***_= the final proportion of the two clones, ***r***_***A ***_= the intrinsic rate of increase of clone ***A ***and ***r***_***a ***_= the intrinsic rate of increase of clone ***a***. Table 2 shows that for small differences in the intrinsic rates of increase between the clones, the number of generations needed for one clone to replace another are very long. However, larger differences in intrinsic rates of increase could result in fairly rapid replacement of one clone by another. In the absence of selection, if two clones differ little in their relative rates of increase, the selective replacement of one clone by another will be slower and therefore there will be more opportunity for additional mutations to arise, resulting in greater clonal heterogeneity.

**Table 2:** Effect of differing relative rates of increase on clonal evolution.

r_A _- r_a_	Number of generations needed for the relative proportion of a clone to change from 1% to 99%
0.01	919

0.05	184

0.10	92

0.50	18

0.75	12

1.00	9

2.00	5

5.00	2

Number of cell generations required in a two clone model for a clone to go from a relative proportion of 1% to a relative proportion of 99% given a range of differences in the intrinsic rate of increase of the two clones. r_A _- r_a _= the intrinsic rate of increase of clone A minus the intrinsic rate of increase of clone a. See text for further explanation.

#### Selection

There are several reasons to expect that competition exists among cancer clones and may in fact be intense. First of all, the environment within a tumour is often characterized by high tumour cell density and areas of tumour cell necrosis suggesting that there are limiting environmental factors to cell survival. Furthermore, hypoxia, host immune responses and therapeutic interventions may create sequential bottlenecks at which selection for the best adapted cancer cell clones could occur [1,3]. The existence of periods of intense selection within a cancer is further supported by finding that tumours may undergo periodic genetic homogenization [33].

Secondly, it is likely that the majority of cancer cells die as a tumour grows. Surgical pathologists regularly see the morphological evidence of this cell death in the form of tumour cell necrosis and apoptotic cells. Additional evidence comes from the apparent mismatch between the mitotic rate and the doubling time of cancers. Doubling time has been measured for a number of different cancers but for illustration I will use the doubling time of lung cancers. Arai et al. [34] measured the mean doubling time of a series of 237 lung cancers as 166.3 days. Table 3 shows the mean number of generations for different tumours with various doubling times. A tumour with a doubling time of 166 days (assuming the rate of growth was constant over a hypothetical 20 year lifespan of the tumour) and no cell death would have experienced an average of 44 cell generations (this is in the same range as the 40 population doublings needed to produce a tumour of maximal size compatible with life [35]. However, if we look at the observed mitotic rates of cancers we would predict a much faster growth rate. For example, in a cancer with a mitotic rate of one mitosis per 1000 cells and assuming a time of mitosis similar to that reported for human epidermis (90 minutes [36]), we would expect a tumour doubling time of 44 days if all cells were dividing at the same rate (Table 3). A mitotic count of one mitosis per 100 tumour cells would give an estimated doubling time of less than 5 days. One mitotic figure count of one per 1000 cells is not an especially high rate considering that one to two mitotic cells per microscopic high power field are commonly encountered in carcinomas and correspond to approximately one mitosis per 300 cells (personal observation). This apparent discrepancy between the observed doubling times and the expected doubling times given a realistic range of observed mitotic rates is easily explained if the vast majority of tumour cells die. This would also allow a much higher number of generations to exist within a cancer than would be predicted solely on the basis of the observed doubling times. For example, in the case of a cancer with 1 in 1000 observed cells in mitosis, 166 generations (not 44) could exist in 20 years if all cells divided at an equal rate. If the tumour contains cancer stem cells, these might be expected to divide at the much faster rate of 50 - 100 generations per year [4], giving 1000-2000 cell generations over the hypothetical 20 year life-span of a tumour. The much larger number of generations present in cancer stem cells would also increase the potential number of mutations which could accumulate in these cells.

**Table 3:** Estimated number of generations over a 20 year period for tumours with different doubling times.

Doubling time (days)	Generations/20 yrs
25	292

50	146

75	97

100	73

125	58

166	44

150	49

200	37

300	24

400	18

500	15

Although the strength of natural selection among tumour cell clones has not been quantified, we can explore the consequences for clonal evolution based on hypothetical levels of selection pressure. Selection can be defined in a number of different ways but for this discussion, I will be referring to selection as the relative advantage of one clone over another. Thus if a given clone is said to have a selective advantage of 0.01, this means that, on average, members of that clone will pass on 1% more offspring to the next generation as compared to a second clone. The change in clonal frequency (expressed as the change in proportion of clone ***A ***to clone ***a ***is described by the equation ***G = [ln(P***_***A***_^***(t)***^***/P***_***a***_^***(t)***^***) - ln(P***_***A***_^***(0)***^***/P***_***a***_^***(0)***^***)]/ln (1+s) ***[22] where ***G ***= the number of generations, ***P***_***A***_^***(0) ***^= the initial proportion of clone ***A***, ***P***_***a***_^***(0) ***^= the initial proportion of clone ***a***, ***P***_***A***_^***(t) ***^= the final proportion of clone ***A***, ***P***_***a***_^***(t) ***^= the final proportion of clone ***a***, and ***s ***= the selective advantage of clone ***A ***over clone ***a***. Analogous to Table 2, Table 4 shows the effect of various selection coefficients on the rate at which one clone can replace another (defined as a change in frequency from 1% to 99%). Table 4 shows that in order for clonal replacement to occur at a rate of one new clone per 30 cell generations (to allow the incorporation of 5 functionally important mutations in 150 cell generations) we would require an ***s ***of slightly less than 0.5. In contrast, for cancer stem cells with 1000 generations, the equivalent number is considerably less, in the range of 0.05 (Table 4). Although a direct comparison would obviously not be valid, it is interesting to note that these levels of selection (0.05 - 0.50) are within the range of selection coefficients observed among species of free-living organisms, where the related measurement of linear selection gradient averages about 0.16 [37]. The necessary level of selection for cancer stem cells (0.05) is, in fact, considerably less than that observed among free-living organisms. Strictly speaking, the equation shown above is applicable only to populations with discrete generations and the more proper comparison of different levels of selection in a population with overlapping generations would be to compare the differences in the intrinsic rates of increase as explained earlier [22]. However, from a practical point of view the two approaches produce similar results and that the equation utilizing the selection coefficient is more instructive in this setting.

**Table 4:** Effect of differing selective advantages on the rate of clonal evolution.

Selective advantage of clone *A *over clone *a*	Number of generations needed for the relative proportion of clone *A *to change from 1% to 99%
0.01	924

0.05	188

0.10	96

0.50	23

0.75	16

1.00	13

2.00	8

5.00	5

### The special case of frequency dependent selection

A final concept from theoretical population genetics which may have some relevance in explaining the behaviour of certain cancers is the concept of frequency-dependent selection [1]. Frequency dependent selection describes the situation where two different species (in our case clones) exist and the fitness of each is greater when it is rare [20]. In the context of tumour biology this could refer to the situation where a cancer contains two different populations - either different clones of the same cell type or entirely different cell types in the case of biphasic neoplasms. Mathematically, this can be described by the equations ***dx/dt = r***_***1***_***x (1-bx-cy) ***and ***dy/dt = r***_***2***_***y (1-fx-gy) ***[20] where ***x ***and ***y ***are the different cell types, ***b ***and ***g ***describe the degree of self-inhibition, ***c ***and ***f ***describe the degree of inhibition of the other cell type, ***r ***is the intrinsic rate of increase of each cell type and ***t ***is the time interval. Depending on the relative values of ***b***,***g***,***c ***and ***f***, it can be shown that the relative density of two types will tend to fluctuate around a stable equilibrium [20], thus maintaining a cancer containing two (or more) distinct cell types. This provides a possible explanation for the existence of biphasic tumours such as synovial sarcomas, the relationship between tumour cells and their surrounding stroma, as well as the existence of the stable long term genetic heterogeneity which has been observed in some cancers [38,39].

## Discussion

In this paper I have attempted to explore some aspects of theoretical population genetics of asexual species and show how these might apply to the population dynamics of cancer cell clones. A few general trends emerge from this exercise. First of all, it is predicted that clonal evolution will be faster (i.e. require fewer generations) when population sizes are smaller. Thus evolution may proceed faster in smaller cancers, in cancers with greater genetic heterogeneity or in cancers in which stem cells are the drivers of clonal evolution. It should also be noted that the models presented here do not specifically address the absolute or relative frequency of cancer stem cells, the only requirement is that they remain less numerous than the non-stem cells. The models presented here also do not address the presence or absence of genetic instability. It is anticipated that genetic instability would increase the mutation rate of all cells and so the predicted relative rates of evolutionary change would remain valid.

The number of generations necessary for mutations to numerically increase in the tumour will depend on either differences in the intrinsic rate of increase of different cancer cell clones or differences in the relative fitness of the clones as measured by relative differences in the selection on each clone. In reality it is likely that a combination of these factors is at work, simultaneously or sequentially, within a tumour. I have given illustrations of the estimated average number of generations for mutations to accumulate within a cancer cell clone given different relative rates of increase and different relative strengths of selection. The major conclusion from these comparisons is that if all somatic cells in a cancer participate in clonal evolution, the number of generations needed to accumulate the necessary number of mutations is within a plausible range, particularly if the number of cell generations within a cancer is greater than previously suspected due to a high cancer cell death rate. If clonal evolution is driven by the accumulation in mutations in cancer stem cells, the differences in intrinsic rates of increase and/or differences in selection need be approximately an order of magnitude less to result in the same rate of clonal evolution.

Whether cancer clones replace each other because of different intrinsic rates of increase or because of differences in the level of selection may be of interest. A possible method to distinguish between these mechanisms is suggested by the prediction that the potentially high level of selection among cancer cell clones would be expected to leave evidence of selection at the genetic level in the form of different rates of synonymous and non-synonymous base substitutions among different clones in the same tumour. The existence of such a difference in substitution rates would provide strong evidence of selection pressure versus a difference in intrinsic rates of increase. This technique may offer a novel and powerful method for detecting positive selection in cancer cell clones. The caveat of course is that one could only comment on the strength of selection acting on the nucleotide sequence in question. Comparison of the relative strength of positive selection in early and late cancers could also provide a way of mapping the relative selection pressures of clones as they evolve. Most importantly, isolation/microdissection of individual stem cells with subsequent genome amplification and sequencing followed by comparison of the relative strength of selection with non-stem cells could furthermore provide a method of testing the relative strength of selection of stem cells and non-stem cells and thereby indirectly testing the entire stem cell paradigm. Finally, it might be assumed that selection pressure acting on cancer cells would be in the form of purifying selection to preserve gene function in the face of the anticipated large number of mutations within a cancer. However, the comparison of the relative rates of synonymous and non-synonymous base substitutions would also allow the detection of diversifying selection (if there was selection to alter the structure of proteins of clones occupying different 'ecological niches' within the cancer).

Additional testable hypotheses suggested by the predictions presented here include the prediction that chemotherapy and surgical debulking of cancers may result in more rapid clonal evolution of the remaining tumour cells.

A major technical hurtle in the further study of clonal dynamics within a cancer has been the lack of an efficient method to estimate intra-tumour clonal diversity, and especially techniques that can be applied to archived clinical samples. A recent advance in this area has been the description of a method to quantify clonal diversity using a combination of FISH and immunofluorescence staining [30]. The application of this or other methods to quantify intra-tumour clonal diversity will lead to an exciting new era in the study of the dynamics of tumour clonal dynamics, and will aid in the testing of the predictions stemming from population genetics.

## Conclusions

Cancer cell clonal evolution has been the subject of intense recent interest; the application of asexual theoretical population genetics to cancer biology has the potential to lead to new insights into this process. The inclusion of cancer stem cells into models of clonal evolution leads to the prediction that in cancers which contain stem cells, clonal evolution of stem cells may be considerably faster than somatic cell evolution.

## Competing interests

The authors declare that they have no competing interests.

## Authors' contributions

This manuscript is the sole work of the submitting author.
